# Managing malaria in the intensive care unit

**DOI:** 10.1093/bja/aeu157

**Published:** 2014-06-19

**Authors:** M. Marks, A. Gupta-Wright, J. F. Doherty, M. Singer, D. Walker

**Affiliations:** 1 The Hospital for Tropical Diseases, Mortimer Market Centre, Capper Street, London, UK; 2 Department of Clinical Research, Faculty of Infectious and Tropical Diseases London School of Hygiene and Tropical Medicine, Keppel Street, London, UK; 3 Department of Critical Care, University College London Hospital NHS Foundation Trust, 3rd Floor, 235 Euston Road, London NW1 2BU, UK

**Keywords:** ARDS, ICU, imported infections, malaria

## Abstract

The number of people travelling to malaria-endemic countries continues to increase, and malaria remains the commonest cause of serious imported infection in non-endemic areas. Severe malaria, mostly caused by *Plasmodium falciparum*, often requires intensive care unit (ICU) admission and can be complicated by cerebral malaria, respiratory distress, acute kidney injury, bleeding complications, and co-infection. The mortality from imported malaria remains significant. This article reviews the manifestations, complications and principles of management of severe malaria as relevant to critical care clinicians, incorporating recent studies of anti-malarial and adjunctive treatment. Effective management of severe malaria includes prompt diagnosis and early institution of effective anti-malarial therapy, recognition of complications, and appropriate supportive management in an ICU. All cases should be discussed with a specialist unit and transfer of the patient considered.

**Editor's key points**

- The authors describe the presentation and management of malaria on the ICU.
- They review the literature and provide management strategies for dealing with this life-threatening condition.

The number of people who travel to malaria-endemic areas continues to increase (Fig. 1).^1^ Malaria is responsible for the death of at least three quarters of a million people worldwide every year^2^ and is the commonest cause of serious imported infection in non-endemic areas.^3–5^ Severe malaria is mostly caused by *Plasmodium falciparum*, although other species can cause severe disease.^6,7^

**Fig 1:**
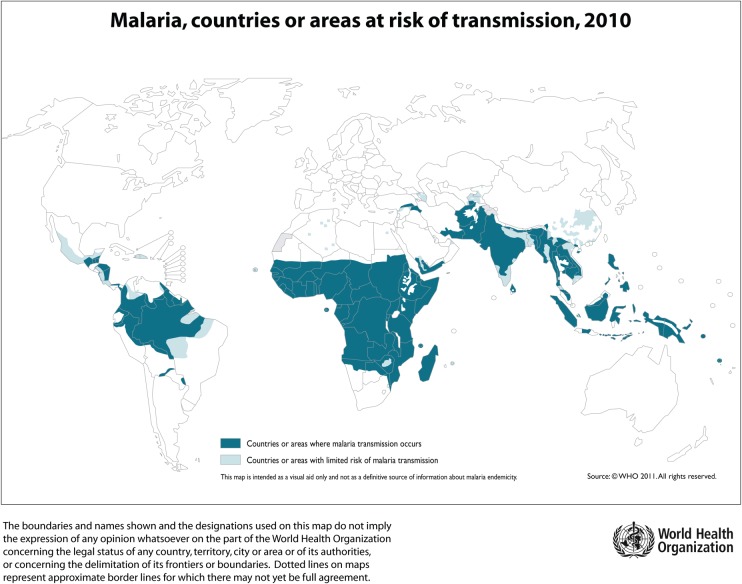
Countries and areas with risk of malaria transmission. Map from WHO International Travel and Health Programme, http://www.who.int/ith/en/. Reproduced with permission of the World Health Organisation.

Recommendations for the management of severe imported malaria are largely derived from trials in endemic regions and retrospective series of imported malaria. In spite of advances in management, the mortality rate of severe malaria remains ∼10% and data from the UK suggest that the outcome may be worse for patients managed in centres with less experience of treating the disease.^8^ This review is based on recent studies of anti-malarial and supportive therapies and outlines the epidemiology of malaria, clinical manifestations, and risk stratification of severe disease, and provides an update on the management of patients with imported malaria requiring intensive care unit (ICU) support.

## Epidemiology

Malaria is endemic throughout most of the tropics and sub-tropics and is one of the commonest causes of febrile illness in returning travellers.^3–5^ There were 6749 cases of imported malaria reported within the European Union in 2010 (0.99 cases per 100 000)^9^ and 1688 cases reported in the USA (0.55 cases per 100 000).^3^ In Europe, four countries (France, UK, Germany, and Italy) account for 80% of all cases. Surveillance from both Europe and the USA show that most cases of falciparum malaria are acquired in sub-Saharan Africa.^3,10–12^ Compared with malaria in endemic settings, where children are most commonly affected, imported malaria is predominantly a disease of young- and middle-aged adults—the median age of cases in the UK is 31 years.^13^

Surveillance data demonstrate that individuals originating from endemic regions who travel to ‘visit friends and relatives’ are more likely to develop malaria than people who travel for other reasons (relative risk 3.65),^5,13^ although these individuals may be at reduced risk of developing severe disease because of partial immunity.^11,14^ Anti-malarial chemoprophylaxis is very effective but surveillance data consistently demonstrate that most travellers do not take it appropriately.^3,13,15^ Reasons for poor adherence include an assumption of low risk, particularly among individuals who grew up in endemic regions, and concerns about potential drug side effects.

Nearly all severe disease is caused by falciparum malaria; ∼10% of patients with imported falciparum malaria are reported to develop severe disease.^3,16^ The case fatality rate is ∼1%.^3,13^ UK surveillance data demonstrate significantly higher mortality (odds ratio 10.68) in patients aged >65 yr compared with adults aged 18–35 yr and among tourists compared with patients originally from endemic countries (odds ratio 8.2).^8^ Death from non-falciparum malaria is extremely rare with a case fatality rate of 0.05%.^8^

## Pathophysiology

**Fig 2:**
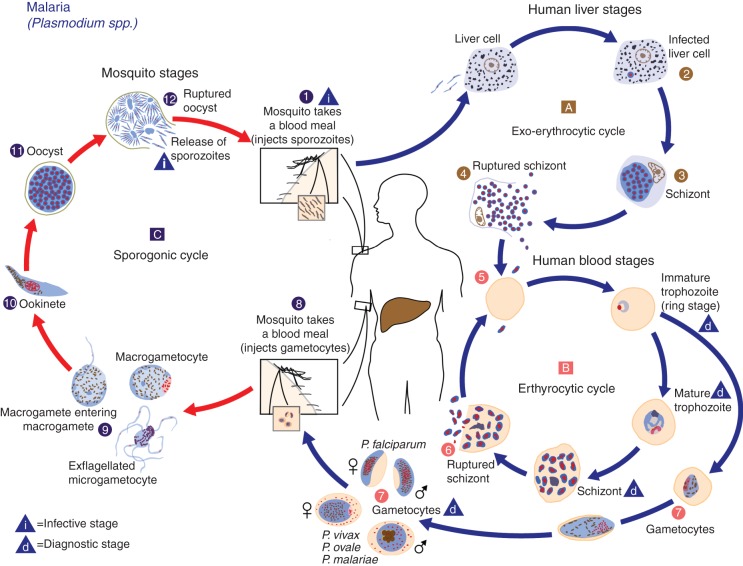
Life cycle of *Plasmodium*. Image from the Centers for Disease Control and Prevention (www.cdc.gov). Image produced by CDC — DPDx/Alexander J. da Silva, Melanie Moser.

Malaria is caused by infection with the protozoan parasite *Plasmodium* and is transmitted by female Anopheline mosquitoes.^17^ Four species are classically considered to cause disease in humans (*P. falciparum*, *Plasmodium vivax*, *P. ovale*, and *P. malariae*) although a fifth, *P. knowlesi*, is now recognized as a zoonotic cause of malaria in parts of Malaysia.^18^ After the bite of an infected Anopheline mosquito (Fig. 2), the inoculated sporozoites are taken up by hepatocytes where they mature over 7–10 days to form schizonts. These then rupture to release variable numbers of merozoites into the blood. Merozoites rapidly invade erythrocytes, forming trophozoites, which again mature into schizonts over a period of 24–72 h, depending on the species. The mature schizonts then rupture causing haemolysis, releasing further merozoites into the blood where they invade more erythrocytes. With *P. falciparum*, each schizont that ruptures releases 16 merozoites into the blood. Most schizonts adhere to the lining of small blood vessels in deep tissues, a process known as sequestration. The presence of schizonts in peripheral blood implies that the parasitaemia is likely to increase significantly and is itself a marker of severe disease. Human disease is caused by these asexual stages. Gametocytes, the sexual stage, develop some days later and it is these that are taken up by mosquitoes in endemic areas, where they breed and multiply in the mid-gut, ultimately leading to sprozoites found in the mosquitoes’ salivary glands. Gametocytes are frequently seen on blood films but, by themselves, are of no clinical significance.^19^

The incubation period for falciparum malaria is usually 12–14 days and slightly longer for non-falciparum species. One series of imported malaria reported a median of 9.5 days (IQR 3–14) between return from a malaria-endemic area and hospital admission.^11^ Progression to severe disease is variable; however, the largest series of severe imported malaria found a mean duration of symptoms of 5.5 days before ICU admission.^20^

Infection with *P. falciparum* results in the expression of *P. falciparum* erythrocyte membrane protein 1 (*Pf*emp1), an important virulence factor, on the surface of red blood cells. *Pf*emp1 mediates binding of infected red blood cells to endothelial surfaces and sequestration in capillary beds.^21^ *Pf*emp1 is encoded by the *var* family of genes and the parasite regularly switches between ∼60 variants of this gene resulting in significant antigenic variation and an impaired immune response.^21^ Release of pro-inflammatory cytokines, including TNF-α and IL-1, in response to infection leads to an up-regulation of endothelial receptors including Intracellular-Adhesion Molecule 1 resulting in further sequestration.^22^ Occlusion of capillary beds leads to microvascular obstruction, tissue hypoperfusion, and lactic acidosis.^23^ Capillary sequestration also impairs splenic clearance of infected red blood cells. The severity of disease is associated with both a higher total body parasite biomass and a higher biomass of sequestered parasites.^24^

## Diagnosis

**Fig 3:**
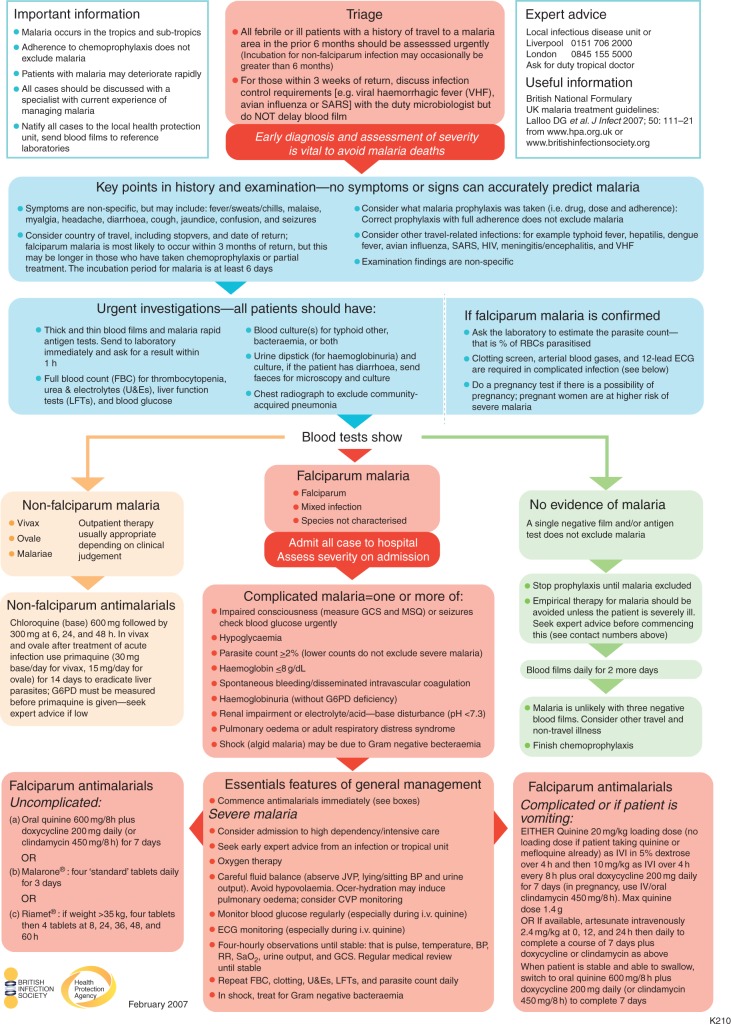
Health Protection Agency and British Infection Associated Algorithm for Initial Assessment and Management of Malaria in Adults.^25^ © Crown copyright. Reproduced with permission of Public Health England.

Microscopic examination of a blood film remains the gold standard for diagnosis of malaria (Fig. 3).^26^ This allows speciation, quantification of parasitaemia, and detection of other markers of severity such as the presence of schizonts. In a non-endemic setting, a parasite count >2% of infected red cells is usually considered a marker of severe disease although lower counts do not exclude this.^26,27^ Both thick and thin films should be examined. Thick films have a higher sensitivity for diagnosis while thin films allow more accurate speciation and quantification of parasitaemia.^28^ Rapid diagnostic tests (RDTs) are used in many settings. RDTs detect circulating parasite-associated proteins and enzymes. Most tests detect both a pan-species target and a falciparum-specific target. RDTs allow diagnosis of malaria without a trained microscopist but do not usually provide speciation nor quantification of parasitaemia. As such, these tests should be considered an adjunct rather than a replacement for blood film analysis.^26,27^ The use of PCR to diagnose malaria remains a research tool, but may occasionally be useful, particularly when there is doubt about the infecting species, for example in the differentiation of *P. knowlesii* from *P. malariae*.

## Criteria for severe or complicated malaria

Criteria for severe malaria in both endemic and non-endemic settings have been defined by the World Health Organisation (WHO) (Table 1).^29^ In imported malaria, the commonest reasons for ICU admission are cerebral malaria, acute respiratory distress syndrome (ARDS), and acute kidney injury (AKI), either alone or in combination.^11,20,30^ As parasitized erythrocytes sequester in deep capillaries, the peripheral parasitaemia may not accurately reflect the true burden of infection. Hyperparasitaemia is therefore not a consistent finding in patients with severe malaria, and may not be a feature in a substantial proportion of patients requiring ICU admission.^11^

**Table 1:** Criteria for severe malaria. Adapted from WHO Guidelines for the treatment of malaria, 2nd Edn.^27^ http://www.who.int/malaria/publications/atoz/9789241547925/en/index.html. Reproduced with permission of the World Health Organisation

**Clinical features of severe falciparum infection**
Cerebral malaria as characterized by impaired consciousness or coma, convulsions, or both
Acute respiratory distress syndrome
Circulatory collapse
Jaundice in the setting of other organ dysfunction
Haemoglobinuria
Abnormal spontaneous bleeding
**Laboratory features of severe falciparum infection**
Hypoglycaemia [<2.2 mmol litre^−1^ (<40 mg dl^−1^)]
Severe anaemia (Hb <5 g dl^−1^, packed cell volume <15%)
Metabolic acidosis (plasma bicarbonate <15 mmol litre^−1^ or pH <7.35)
Hyperparasitaemia (>2%/100 000 µl^−1^ in low-intensity transmission areas or >5% or 250 000 µl^−1^ in areas of high stable malaria transmission intensity)
Hyperlactataemia (lactate >5 mmol litre^−1^)
Acute kidney injury (serum creatinine >265 µmol litre^−1^).

## Differential diagnosis

The differential diagnosis of severe malaria is broad and varies depending on the patient's travel history.^31^ Major considerations include the common causes of community-acquired Gram-positive and Gram-negative bacterial sepsis, enteric fever, severe rickettsial infections, and leptospirosis, and also arboviral infections (including dengue fever) and the viral haemorrhagic fevers. Consultation with a specialist is recommended if there is any doubt as to the diagnoses or in any patient considered to be at risk of viral haemorrhagic fever.

## Anti-malarial drugs

Since the emergence of chloroquine resistance in the 1970s, parenteral quinine has been the mainstay of treatment for severe malaria (Table 2). The commonest dose-related side effect of quinine is *cinchonism*, which comprises tinnitus, blurred vision, reversible hearing loss, headache, and nausea. Quinine may also cause hypoglycaemia and prolongation of the QTc interval on an electroencephalography (ECG) necessitating regular monitoring, but is usually well tolerated. Artemisinins, derived from the Chinese herb *qinghausu* or wormwood, have been used by Chinese traditional healers for many years but have become available to western practioners only in recent years.

**Table 2:** Anti-malarial therapy at the Hospital for Tropical Diseases. The Hospital for Tropical Diseases' treatment guidelines are based on part of the UK malaria treatment guidelines^26^ but have been updated to reflect the growing importance of artesunate since the guidelines were published. *Quinine therapy should not be delayed if artesunate is not immediately available. Patients do not require treatment with both artesunate and quinine. Once the first dose of artesunate has been given, quinine can be stopped

**Route of administration**	**Quinine**	Artesunate*
	**i.v.**	**i.v.**
Dosing	20 mg kg^−1^ loading dose (max 1400 mg) Subsequently 10 mg kg^−1^ (max 700 mg) given 8 hourly	2.4 mg kg^−1^ at 0, 12, and 24 h and then once daily
Side effects	Cinchonism—tinnitus, visual blurring, and nausea. Reversible and not an indication to stop quinine Hypoglycaemia Prolongation of the QT interval	Normally well tolerated although posttreatment haemolysis is recognized
Monitoring	Capillary blood sugar 2–4 h ECG monitoring of QTc Continuous cardiac monitoring advised in patients with underlying cardiac disease	Not required
Follow-on therapy Therapy can be switched once the patient is improving clinically, the parasite count is <2%, and they can tolerate oral medication. Discuss with an expert	Oral quinine 10 mg kg^−1^ (max 700 mg) TDS to complete 7 days total course with either Doxycycline 200 mg for 1 week or Clindamycin 450 mg TDS for 1 week Doxycycline of clindamycin can be given either simultaneously (with both i.v./oral quinine) or after completion of quinine therapy	Artemether/Lumefantrine (Riamet, Co-Artem) four tablets at 0, 8, 24, 36, 48, and 60 h. Quinine (10 mg kg^−1^ max 700 mg) with doxycycline or clindamycin for a total of 7 days Atovaquone/Proguanil (Malarone) four tablets OD for 3 days

The artemisinin derivative artesunate has recently been shown to be more effective than quinine for the treatment of severe falciparum malaria in two large randomized trials.^32,33^ The SEAQUAMAT study randomized 1461 mostly adult patients with severe malaria in South and South-East Asia, and showed a significant reduction in mortality in patients treated with artesunate compared with quinine (15% *vs* 22%, *P*=0.0002).^32^ The subsequent AQUAMAT study randomized 5425 children (<15 years) with severe malaria across eleven countries in sub-Saharan Africa and again showed a significant reduction in mortality with artesunate (8.5% *vs* 10.9% for quinine, *P*=0.0022).^33^ Routine monitoring for hypoglycaemia and QT prolongation are unnecessary if an artemisinin derivative is used. Although extremely effective in treating malaria, they cannot be used as single agents as the rate of recrudescent infection is very high.

Availability of artesunate has been limited in many countries, including the UK, as the drug is not licenced and is only available on a named patient basis.^34^ Because of this, parenteral artesunate is only available in relatively few specialist centres. Treatment with quinine should not be delayed while artesunate is obtained. If there is any doubt about the infecting species, then treatment for falciparum should be prescribed. Artemisinin combination treatments (ACTs), which combine an artemisinin with another effective anti-malarial drug, are now recommended by the WHO as first-line treatment for non-severe malaria^27^ and should be used after initial treatment with parental artesunate. Early liaison with a specialist tropical medicine or infectious diseases unit is essential to ensure appropriate treatment as soon as possible. The treatment strategy used at the Hospital for Tropical Diseases, London, UK, is outlined in Table 2.

Resistance to quinine has been widely reported from South-East Asia and occasionally from sub-Saharan Africa.^35^ However, in the context of malaria, resistance is a relative rather than an absolute phenomenon. Partial artemisinin resistance has also been seen in South-East Asia, especially Cambodia, and Thailand^36–38^ and results in a decreased rate of clearance of asexual parasites from the blood. Treatment failure with ACTs has not yet been reported, but its development and the potential for artemisinin resistance spreading to Africa is a major concern.

## Exchange transfusion

Several anecdotal reports and some case series have supported the use of exchange transfusion in severe malaria, especially in patients with high parasitaemia; it continues to be recommended in some national guidelines.^26^ The rationale is that exchange transfusion removes both infected red cells, lowering parasite burden and parasite-derived antigen load, and circulating pro-inflammatory cytokines. A recent report from a single-centre reported no deaths among 25 patients who had an exchange performed.^39^ However, a meta-analysis in 2002 found only eight quasi-experimental studies of the efficacy of exchange transfusion and demonstrated no overall survival benefit, a finding echoed in a recent study of US patients;^40^ the WHO states that no recommendation regarding exchange transfusion is possible based on the paucity of current evidence.^27,41^ At our centre, exchange transfusion has not been used since the introduction of artemisinin-based therapy for all patients with severe malaria.

## Fluids and cardiac function

The traditional adage is that patients with severe malaria should be managed with relatively restricted fluid volumes because of concerns about the risk of capillary leak and lung and cerebral oedema. This practice is supported by the movement in critical care to a more fluid restrictive practice in patients with acute lung injury (ALI).^42^ However, this restrictive fluid regimen commences only after initial resuscitation has been completed. No large trials have assessed fluid resuscitation strategies in patients with severe malaria in a developed world ICU setting; current data are derived only from trials in endemic areas and from small physiological studies.

The fluid expansion as supportive therapy study randomized 3141 African children presenting with severe febrile illness to maintenance fluids with or without boluses of either crystalloid (0.9% saline) or colloid (5% human albumin solution).^43^ Malaria was the reason for admission in 57% of these children. The trial was stopped early because of a significant increase in mortality in both groups who received fluid boluses compared with the group receiving maintenance fluids only (4 week mortality 8.7% *vs* 12.0% and 12.2%, *P*=0.004 for comparison of maintenance with bolus). In a *post hoc* analysis, there was an increase in terminal cardiovascular events related to fluid resuscitation.^44^ There were no differences in mortality between the sub-groups of children with malarial and bacterial infections. There are obvious limitations in applying these findings to critical care settings in the developed world as the children could not be offered mechanical ventilation, renal replacement therapy, nor inotropic support. Furthermore, time to hospital presentation, while not formally measured, was likely to be significantly longer than in a developed world setting, so the potential risk of an exaggerated reperfusion injury after aggressive resuscitation cannot be discounted.

Although not considered a traditional manifestation of severe malaria, there is emerging evidence that cardiac dysfunction may complicate severe disease. A number of studies have found evidence of increased circulating levels of cardiac enzymes including BNP in individuals with severe malaria.^45–47^ Intravascular haemolysis as a result of severe malaria has been shown in one small study to result in decreased levels of nitric oxide,^47^ increased pulmonary pressures, and myocardial wall stress. Further studies to understand the clinical importance of cardiac dysfunction in severe malaria are warranted.

Previous studies have demonstrated that traditional markers of fluid balance correlate poorly with acid–base status and respiratory function.^48^ The argument against aggressive fluid loading is strengthened by recent data on the physiological response of 28 adult ICU patients with severe malaria to fluid expansion guided by invasive cardiac monitoring.^23^ Despite trans-pulmonary thermodilution (PiCCO) guided therapy, acid–base status deteriorated in 68% and no improvement in renal function was observed after volume expansion. Significant increases in extravascular lung water occurred in 17 of 22 (77%) patients who were liberally resuscitated, with eight developing frank pulmonary oedema despite being hypo- or euvolaemic. Five patients died, all of whom developed pulmonary oedema. The authors found that the degree of lactataemia correlated with the degree of parasite microvascular sequestration, but not with hypovolaemia.

It would appear that, in the absence of prospective, randomized trial data, liberal fluid therapy is best avoided in the context of severe malaria. Because of the propensity of these patients towards capillary leak and thus a greater risk of ARDS and cerebral oedema, our own management approach is to target significant hypovolaemia with concurrent tissue hypoperfusion, that is, clinical markers of organ dysfunction (e.g. oliguria) with biochemical markers (e.g. lactate and central venous oxygen saturation). Fluid loading is guided by a goal-directed algorithm using both the stroke volume response to a fluid challenge (measured by minimally invasive oesophageal Doppler) and markers of tissue perfusion as therapeutic endpoints. Otherwise, fluid balance is kept neutral, in line with studies in critically ill patients showing a strong association between positive fluid balance and worse outcomes.^49^ Where vasopressor, inotrope, or both requirements persist, we maintain an acceptable cardiac output, avoiding fluid overload but targeting tissue perfusion and have a low threshold for diuresis in the haemodynamically stable patient in the postinflammatory phase of illness.

## Respiratory manifestations

The WHO defines respiratory manifestations of severe malaria in terms of deep breathing, respiratory distress, and pulmonary oedema.^27^ Cough is a common symptom,^50^ and tachypnoea may be caused by fever, anaemia, and a metabolic acidosis, and also primary lung pathology such as the ARDS and pneumonia. ARDS has clear diagnostic criteria that have been recently redefined,^51^ with the previous clinical distinction between ALI and ARDS being replaced by mild, moderate, and severe levels of ARDS. This condition is more common among adults than children.^52^ The reported incidence of ‘respiratory distress’ in severe malaria varies between 2 and 30%, with differences in definitions accounting for some of this variation.^10–12,20,52–54^ ARDS and respiratory distress are poor prognostic signs in both endemic and imported malaria.^20,55,56^

The mechanisms underlying ARDS are not entirely understood, but likely causes include endothelial dysfunction and altered capillary permeability because of parasitized erythrocyte adherence and sequestration and exaggerated host immune and inflammatory responses, particularly TNF-α, IL-1, IL-6, and IL-8.^57,58^ However, ARDS can develop after apparently successful treatment and after the disappearance of parasites from the blood.^11,53^ In these cases, ARDS may reflect persistence of inflammatory cytokines in the absence of any infected erythrocytes. There is emerging evidence that free parasite antigens may persist after treatment suggesting that these may represent a potential on-going stimuli for inflammation.^52^ Concurrent bacterial pneumonia and cardiogenic pulmonary oedema (which may be iatrogenic, the result of renal failure, severe anaemia, or heart failure related to a sepsis-induced myocardial depression) are other important causes of respiratory distress.

As there are no specific trials addressing ARDS treatment in malaria, strategies are based on evidence-based ARDS management, including the use of low tidal volume ‘protective’ ventilation and moderate levels of PEEP.^59–62^ Fluid balance is kept neutral, or negative if the patient is considered to be volume overloaded. Cerebral oedema and raised intracranial pressure associated with cerebral malaria may limit permissive hypercapnia and the use of high PEEP strategies; however, pragmatic clinical decision-making should be used.^63^ Successful use of extracorporeal membrane oxygenation for severe respiratory failure has been reported in malaria.^12,64^ Bacterial co-infection is relatively common suggesting that a low threshold for starting antibiotics, when supported by clinical and laboratory investigations, may be appropriate.^11,20,30^

## Hypoglycaemia

Hypoglycaemia, defined as blood glucose values <2.2 mmol litre^−1^ (<40 mg dl^−1^),^27^ is a common complication of malaria and can be a marker of severe disease, particularly in children.^65,66^ Case series of imported malaria report a prevalence of hypoglycaemia between 1 and 20% at admission, with a higher rate among those who die.^10–12,20,54^ The pathogenesis is poorly understood but is thought to be related both to parasite glucose consumption and to impaired host gluconeogenesis rather than to malnutrition or hyper-insulinaemia.^67^ Hypoglycaemia may be exacerbated by parenteral quinine (an insulin secretagogue). A meta-analysis reported a significantly lower incidence during treatment with artemisinins compared with quinine [combined HR 0.55 (95% CI 0.41–0.74)].^68^ Clinical features include a reduced level of consciousness and seizures. Blood glucose should be routinely and regularly assessed and monitored, especially during treatment with quinine. Early enteral feeding has been established as beneficial in a wide range of patients requiring intensive care, and may mitigate against hypoglycaemia in severe malaria.^69,70^

## Neurological involvement

Cerebral malaria is strictly defined as coma [Glasgow Coma Score (GCS) <9] in a patient with malaria in whom other aetiologies have been excluded.^29^ In clinical practice, a decrease in GCS <11 or the occurrence of seizures should be taken to represent cerebral malaria once hypoglycaemia and other potential causes of reduced consciousness have been excluded. As with respiratory distress, cerebral malaria is associated with worse outcomes.^71^ The pathogenesis remains incompletely understood.^72^ Electroencephalography has previously found sub-clinical seizure activity in a proportion of patients with cerebral malaria^73^ which prompted three anti-epileptic trials.^74–76^ However, despite a reduction in seizure frequency, mortality was increased in patients receiving routine anti-convulsant therapy with phenobarbital.^77^ This increased mortality is postulated to occur as a result of respiratory depression. The extent to which these findings can be generalized to other classes of anti-epileptics, in particular those that cause less respiratory depression, is unknown. In view of these findings, there is currently no role for routine EEG monitoring and the use of anti-epileptics in patients with cerebral malaria should be limited to those with clinically overt seizure activity.

Cerebral oedema is a well-recognized component of cerebral malaria and strategies to reduce this have been assessed in controlled trials. Warrell and colleagues^78^ randomized patients with cerebral malaria to receive either dexamethasone or placebo. Mortality did not differ between the two groups (*P*=0.8) but coma was prolonged in those who received dexamethasone [63.2 (5.9) h *vs* 47.4 (3.2) h, *P*=0.02]. This finding is consistent with other studies of corticosteroids in cerebral oedema because of other aetiologies such as head injury.^79^ Complications, including gastrointestinal bleeding and pneumonia, were also more common in patients receiving dexamethasone (*P*=0.004). Mohanty and colleagues^80^ randomized 61 adult patients with cerebral malaria and CT-confirmed cerebral oedema to adjunctive treatment with mannitol. There was a non-significant trend towards a higher mortality in those receiving mannitol (30% *vs* 13%, HR 2.4 95% CI 0.8–7.3, *P*=0.11). Mannitol was also associated with a significant increase in the duration of coma (90 h compared with 32 h with placebo, *P*=0.02). Other adjunctive treatments including N-acetyl cysteine, heparin, aspirin, deferoxamine, anti-TNF therapy, and pentoxifylline have all been trialled but none has been shown to be of benefit.^81–85^ No adjunctive treatments are currently recommended for cerebral malaria.

## Acute kidney injury

AKI in malaria is usually caused by *P. falciparum*, although it has been reported with other species.^86,87^ The WHO uses a serum creatinine of >265 µmol litre^−1^ (or ≥3 mg dl^−1^) as a criterion for severe malaria,^27^ although this definition is at variance with commonly applied definitions of AKI.^88^ AKI is particularly common among individuals who did not grow up in endemic regions, suggesting that it may be more common in the malaria-naïve.^89–91^ The incidence of AKI in severe malaria varies from 1 to 5% in endemic areas,^91^ but the rate of AKI is much higher in series of imported malaria (ranging from 23 to >50%).^10–12,20,53,54^ Cytoadherence of parasitized erythrocytes to glomerular and tubular vascular beds, cytokine release, immune complex deposition, hypovolaemia, and haemolysis may all be contributory.^57,91–93^ Histopathological findings of AKI in severe malaria include acute tubular necrosis, interstitial nephritis, and glomerulonephritis, although tubular changes are the most common findings.^89^

All patients with falciparum malaria should be screened for AKI, which may not develop until several days after the onset of fever and can be non-oliguric.^91^ Management is supportive with maintenance of fluid balance and electrolytes and renal replacement therapy as indicated. Trials of both dopamine and epinephrine have been performed in severe malaria, but neither has been shown to improve renal oxygen metabolism nor function.^94^ Artemisinin doses do not need adjusting in AKI; however, quinine may accumulate, so doses should be reduced by one-third after 48 h of established renal failure, unless renal replacement therapy has been initiated.^27^ The prognosis of AKI associated with severe malaria is usually good, and it inevitably resolves in days to weeks. A recent UK series found that even those patients with persisting renal impairment at discharge from ICU ultimately recovered their renal function.^11^

## Co-infection

In endemic areas, concurrent community-acquired Gram-negative bacteraemia, in particular with non-typhoidal *Salmonellae*, has been shown to occur in 5–12% of children with malaria.^95–97^ Recent studies have suggested that almost two-thirds of cases of community bacteraemia in endemic regions may be the result of malaria. Furthermore, invasive bacterial disease is associated with a worse prognosis.^95–97^ One proposed mechanism is induction of heme oxygenase-1, which mediates tolerance to malaria-induced haemolysis, resulting in reduced resistance to infection with non-typhoidal *Salmonellae.*^98^ However, few data are available on the frequency of bacteraemia in adult patients or returned travellers.

Rates of microbiologically confirmed community-acquired bacterial infections have been reported as 5–10% in patients with imported malaria requiring ICU admission.^11,20,30^ Pneumonia was the commonest co-infection. Community-acquired bacteraemia has been reported in 1.5–3% of cases requiring ICU admission. However, these studies were all retrospective and a failure to take blood cultures before antibiotics were administered may have resulted in an under-estimate of the true frequency of bacteraemia. The possibility of co-infection in returning travellers is an additional reason for early liaison with a specialist unit. High rates of co-infection with HIV have also been reported in some series.^11^

The use of empiric antibiotics remains controversial, but bacterial co-infection should be suspected in any patient with focal signs or symptoms of sepsis or significant neutrophilia. In such cases, blood cultures should be taken and broad-spectrum antibiotics commenced,^27^ albeit de-escalating quickly or stopping treatment if bacterial infection is subsequently not confirmed. Clinicians should also remain alert to the possibility of nosocomial infection. Ventilator-associated pneumonia and catheter-related sepsis are well recognized in this setting and frequently contribute to poor outcomes despite adequate anti-malarial treatment.^11,20^

## Anaemia and coagulopathy

Malarial anaemia is caused by a combination of haemolysis, dyserythropoiesis, and removal of infected erythrocytes from the circulation by the spleen. Parasite antigens, antibody activation, and subtle alterations in red cell membranes may also result in a similar fate for uninfected cells.^99^ WHO defines severe anaemia as a haemoglobin concentration of <5 g dl^−1^.^27^ However, severe malarial anaemia is mostly seen in endemic areas, especially among children and pregnant women, and is likely to be multifactorial.^99^ Only one series of imported malaria reported patients presenting with severe anaemia as defined by the WHO.^20^ Transfusion is recommended in severe anaemia, although no specific studies have addressed transfusion targets in malaria. Current critical care practice supports a restrictive use of red cell transfusions, and this is supported by WHO recommendations of a haemoglobin threshold of 7 g dl^−1^, taking into account individual clinical circumstances.^27,100,101^

Clinically apparent abnormal bleeding and coagulopathy is commonly seen in severe imported malaria, with a reported frequency ranging from <5%^20,54,102,103^ to 20% or more.^10–12,53^ Profound thrombocytopenia is common in both severe and non-severe falciparum malaria and is probably caused by increased platelet consumption, sequestration within the spleen, or both. Disseminated intravascular coagulation, which occurs in about 5–10% of severe imported malaria, should be treated conventionally with transfusion of screened blood products (whole blood, cryoprecipitate, fresh frozen plasma, and platelets) and guided by haematological expertise, but there is no evidence to support empirical platelet transfusion.^11,12,20^

## Prognosis

Mortality from severe malaria varies enormously by setting and clinical context. A mortality of >30% has been reported in children with respiratory distress and impaired consciousness.^55^ In the UK, the overall case fatality rate from falciparum malaria is ∼1%.^8^ Increased age and management at a centre with less experience of managing malaria have both been identified as risk factors for increased mortality, while individuals born in endemic countries had a lower mortality.^8^ In studies of artemisinins, overall mortality was 10–19%.^32,33^ Case series of patients with imported malaria requiring ICU admission have reported mortality rates between 5 and 29%.^11,104^ Older age, reduced GCS, and higher parasitaemia at ICU admission were significantly associated with an increased mortality in the largest cohort,^20^ albeit not consistently replicated in other studies.^11,30^

## Risk stratification

Two scoring systems have been proposed for the stratification of adult patients with severe malaria.^105,106^ Hanson and colleagues^105^ derived a simple score (coma–acidosis–malaria score) using arterial base deficit and GCS derived from SEAQUAMAT trial data.^32^ Patients score 0–2 points for their GCS level and 0–2 points for base deficit. A total score of <2 accurately identified patients who survived (positive predictive value 95.8%). However, the positive predictive value of CAM scores for mortality is more limited.

Mishra and colleagues^106^ derived the malaria score for adults (MSA) based on the presence or absence of severe anaemia (1 point), AKI (2 points), respiratory distress (3 points), and cerebral malaria (4 points). Mortality increased steadily with an increasing MSA, from 2% for MSA scores of 0–2 to 90% for those scoring ≥7. Taking MSA scores of 5 as a cut-off, they reported a sensitivity of 89.9% for mortality and a positive predictive value of 94.1%. As with the CAM score, patients with imported malaria having a low score (<5) had good predictive power for survival, whereas high scores had a limited predictive power for death.^11^ The utility of both these scores is likely to vary significantly between resource-rich and resource-scarce settings.

## Conclusions

Rates of international travel continue to increase and the ‘febrile returned traveller’ is an increasingly common clinical problem. Malaria remains the most important cause of imported fever and cases requiring ICU admission continue to be associated with a high mortality. While there have been significant advances in our understanding of the management of malaria in the last decade, high-quality data to guide management of imported malaria remain scarce, with most derived from endemic settings or retrospective series. The emergence of artemisinin-based therapy has translated into a significant improvement in outcomes in endemic countries and is likely to improve outcomes in imported malaria in the future. Despite numerous studies, no adjunctive therapy has been shown yet to confer a survival advantage and several have proved harmful. ICU management remains supportive and improved outcomes may be attributable more to advances in multi-disciplinary team working, mechanical ventilation strategies, careful fluid management, and infection control. Despite these advances, the mortality from imported malaria remains significant; all cases should be discussed with a specialist unit and transfer of the patient considered.

## Authors’ contributions

M.M. and A.G.-W. wrote the first and subsequent drafts of the paper. M.S. reviewed and redrafted the paper. J.F.D. and D.W. conceived of the article, reviewed, and redrafted the paper.

## Declaration of interest

The authors declare that they have no relevant conflicts of interest.

## Funding

This study was supported by the Special Trustees of the Hospital for Tropical Diseases. All the authors are supported by the UCLH/UCL Biomedical Research Centre Infection, Immunity and Inflammation Theme. Michael Marks is a Wellcome Trust Clinical Research Fellow (WT102807) at the London School of Hygiene and Tropical Medicine. The funding agencies had no role in study design, data collection and analysis, decision to publish, or preparation of the manuscript.
